# Potential toxicity of graphene (oxide) quantum dots via directly covering the active site of anterior gradient homolog 2 protein

**DOI:** 10.1038/s41598-024-57677-9

**Published:** 2024-03-26

**Authors:** Yuqi Luo, Zonglin Gu, Hailiang Chen, Yaoxing Huang

**Affiliations:** 1 Department of Gastrointestinal and Hepatobiliary Surgery, Shenzhen Longhua District Central Hospital, No. 187, Guanlan Road, Longhua District, Shenzhen, 518110 Guangdong China; 2 College of Physical Science and Technology, Yangzhou University, Jiangsu, 225009 China; 3 Department of Gastroenterology, Guangzhou First People’s Hospital, School of Medicine, South China University of Technology, Guangzhou, 510180 Guangdong China

**Keywords:** Graphene quantum dots, Graphene oxide quantum dots, Anterior gradient homolog 2 protein, Touch and covering, Molecular dynamics simulation, Biophysics, Computational biology and bioinformatics

## Abstract

Graphene quantum dots (GQDs) have attracted significant attention in biomedicine, while extensive investigations have revealed a reverse regarding the potential biotoxicity of GQDs. In order to supplementing the understanding of the toxicity profile of GQDs, this study employs a molecular dynamics (MD) simulation approach to systematically investigate the potential toxicity of both GQDs and Graphene Oxide Quantum Dots (GOQDs) on the Anterior Gradient Homolog 2 (AGR2) protein, a key protein capable of protecting the intestine. We construct two typical simulation systems, in which an AGR2 protein is encircled by either GQDs or GOQDs. The MD results demonstrate that both GQDs and GOQDs can directly make contact with and even cover the active site (specifically, the Cys81 amino acid) of the AGR2 protein. This suggests that GQDs and GOQDs have the capability to inhibit or interfere with the normal biological interaction of the AGR2 active site with its target protein. Thus, GQDs and GOQDs exhibit potential detrimental effects on the AGR2 protein. Detailed analyses reveal that GQDs adhere to the Cys81 residue due to van der Waals (vdW) interaction forces, whereas GOQDs attach to the Cys81 residue through a combination of vdW (primary) and Coulomb (secondary) interactions. Furthermore, GQDs aggregation typically adsorb onto the AGR2 active site, while GOQDs adsorb to the active site of AGR2 one by one. Consequently, these findings shed new light on the potential adverse impact of GQDs and GOQDs on the AGR2 protein via directly covering the active site of AGR2, providing valuable molecular insights for the toxicity profile of GQD nanomaterials.

## Introduction

In recent decades, carbon-based nanomaterials (CBNs) have emerged as a prominent area of scientific research, primarily driven by seminal discoveries of fullerene C60 in 1985, carbon nanotubes (CNTs) in 1991, and graphene in 2004^1–3^. The unique and exceptional properties intrinsic to CBNs, encompassing high specific surface area, size-dependent effects, structural adaptability, and superior mechanical, electrical, and optical traits, have generated considerable interest across diverse scientific communities^4–8^. Consequently, CBNs have found versatile applications as gas storage devices, transistors, sensors, nanocarriers, and nanodrugs^9–13^. Extensive efforts within the biomedical field have been devoted to exploring the applications of CBNs^14–17^. However, prior to their formal utilization, thorough consideration of the potential toxicity of these materials is essential^18,19^. Notably, graphene, a remarkable CBN, exhibits severe toxicity towards specific biomolecules. Research by Tu et al.^20^ has elucidated the pronounced insertion of graphene and graphene oxide into cellular membranes, causing lipid extraction and subsequent cell death. Various studies^21^ have revealed graphene's capability to disrupt the structural integrity of proteins, impacting their secondary and tertiary structures, leading to protein toxicity. Furthermore, graphene has been observed to interfere with physiological protein–protein interactions, thus disrupting signal transduction^22^, and potentially influencing the helical conformation and base pairs of double-stranded DNA, posing risks of genotoxicity^23^.

Due to their diminutive size and quantum effects, graphene quantum dots (GQDs) have garnered significant attention across various scientific fields^24–26^. Notably, the 2023 Nobel Prize in Chemistry was awarded to three scientists for their pioneering discoveries and synthesis of quantum dots, further enhancing the possibility of GQDs as a focal point of future research. In detail, Alexei Ekimov reported size quantization effects in CuCl embedded in glass^27^, Luis Brus prepared colloidal CdS suspensions with the demonstration of size quantization effects^28^, and Moungi Bawendi reported a new procedure to synthesize CdE (E = S, Se, Te) nanocrystals using a hot injection method^29^. In considering the application of GQDs, assessing their biocompatibility in organisms is a pivotal factor^30,31^. Research conducted by Chong et al.^32^ investigated the in vitro and in vivo toxicity of GQDs using a variety of analytical techniques. Their findings demonstrated the minimal cytotoxicity of GQDs owing to their ultra-small size and high oxygen content. Similarly, Chu et al.'s investigations of mammalian reproductive and offspring health indicated no discernible effects on the frequency and timing of sexual behaviors in male mice following exposure to GQDs via oral gavage or intravenous injection^33^. Additionally, Xu et al.'s work^34^ demonstrated weak toxicity of luminescent GQDs towards HeLa cells and zebrafish embryos. Nevertheless, Das et al.^35^ emphasized the nuanced GQD toxicity, highlighting factors such as size, concentration, surface chemistry, and doping as pivotal determinants. Notably, certain studies^36,37^ underscore potential toxic effects, including dopaminergic neurodegeneration, induction of apoptosis, autophagy, and inflammatory responses. Hence, a comprehensive evaluation of the toxicity profile of GQDs is crucial for a thorough understanding of their biosafety.

Some research have showed that low doses of smaller graphene nanosheets (e.g., GQD and GOQD) are able to distribute in living organisms and subsequently become the source of graphene toxicity^38^. Thus, lower doses exhibit greater toxicity compared to higher doses due to the tendency of graphene to accumulate within the digestive system, altering the internal exposure. Of particular significance is the enrichment of the intestine, leading to direct contact between intestinal proteins and GQDs or GOQDs, consequently resulting in potential toxicity. In this context, we have selected a pivotal and relevant protein in the intestine, namely the Anterior Gradient Homolog 2 (AGR2) protein, to explore the potential impact of GQDs and GOQDs on this protein. AGR2 plays a vital role in the in vivo production of the intestinal mucin, a large cysteine-rich glycoprotein responsible for forming the protective mucus gel lining the intestine. Experimental evidence showed that a specific cysteine residue (Cys81) within the AGR2 thioredoxin-like domain is involved in forming mixed disulfide bonds with MUC2, indicating a direct involvement of AGR2 in mucin processing^39^. Studies on mice lacking AGR2 revealed their heightened susceptibility to colitis, confirming the critical role of AGR2 in protecting against disease. In addition, experimental study^40^ has shown that the toxicity graphene is dependent on sizes, that is, graphene with smaller size causes a more serious damage to human cells. Thus, in our theoretical simulations, we use the small sized GQDs, rather than graphene. Our molecular dynamics (MD) results demonstrate that both GQDs and GOQDs can directly adhere to and cover the active site (a Cysteine amino acid) of AGR2, hindering its normal exposure. This interference potentially exerts a detrimental influence on the intestine.

## Results

### GQDs bind to the active site of AGR2 protein

**Figure 1:**
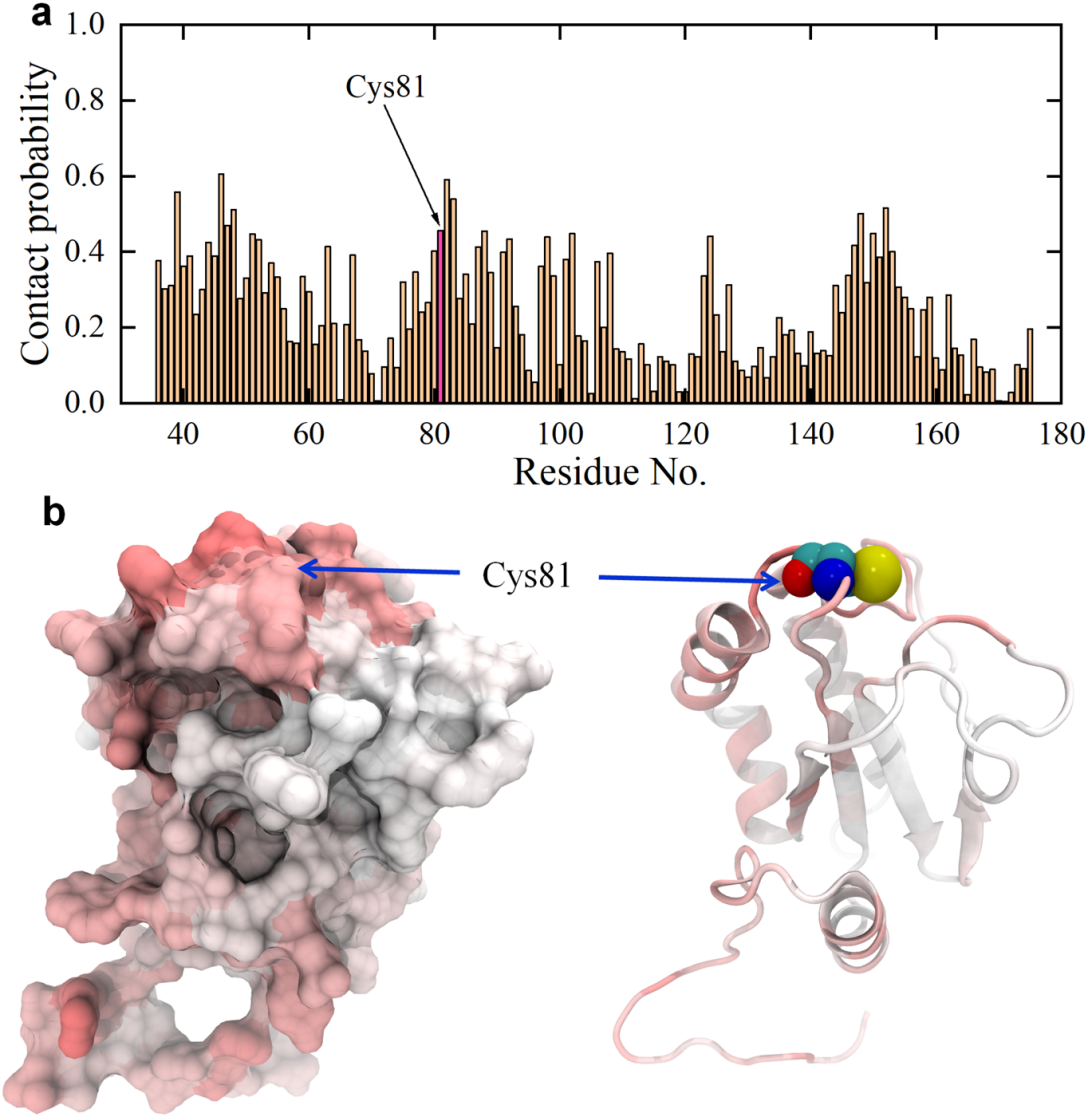
(**a**) Contact probability of GQDs to each residue of AGR2 protein. The data are averaged from ten parallel trajectories. The contact probability of active site of AGR2, Cys81, is highlighted. (**b**) Left: contact probability surface of AGR2; Right: contact probability secondary structure of AGR2. The red and white colors indicate the high and low contact probabilities of GQDs to AGR2 protein. The Cys81 is pointed by blue arrows and is shown with spheres (carbon atoms: cyan; oxygen atom: red; nitrogen atom: blue; sulfur atom: yellow) in the right picture.

Two typical simulation systems (depicted in Fig. S1) were designed to explore the potential impact of GQD and GOQD on AGR2, a notable and pivotal protein in the intestinal environment. Each system performed Molecular Dynamics (MD) simulations along ten parallel trajectories, with each trajectory lasting 100 ns. The initial investigation based on the contact probability of GQDs with each residue of the AGR2 protein, as shown in Fig. 1. Evidently, numerous AGR2 residues exhibit notably high contact probabilities with GQDs, surpassing 0.2. Conversely, only a few residues displayed zero contact probability, indicating a pronounced propensity for GQDs to adhere to the surface of the AGR2 protein. Notably, AGR2 contains a vital active site, Cys81, pivotal in forming mixed disulfide bonds with intestinal mucin MUC2, signifying its significant role in maintaining intestinal health. Therefore, the obstruction of the AGR2 active site might potentially yield adverse effects. Our findings reveal a high contact probability of Cys81 to GQDs, nearing a value of 0.456, indicating GQDs' robust interaction with this residue. Such easy interaction between GQDs and Cys81 might lead to potential inhibition of AGR2's association with its target protein, potentially inducing toxic effects.

**Figure 2:**
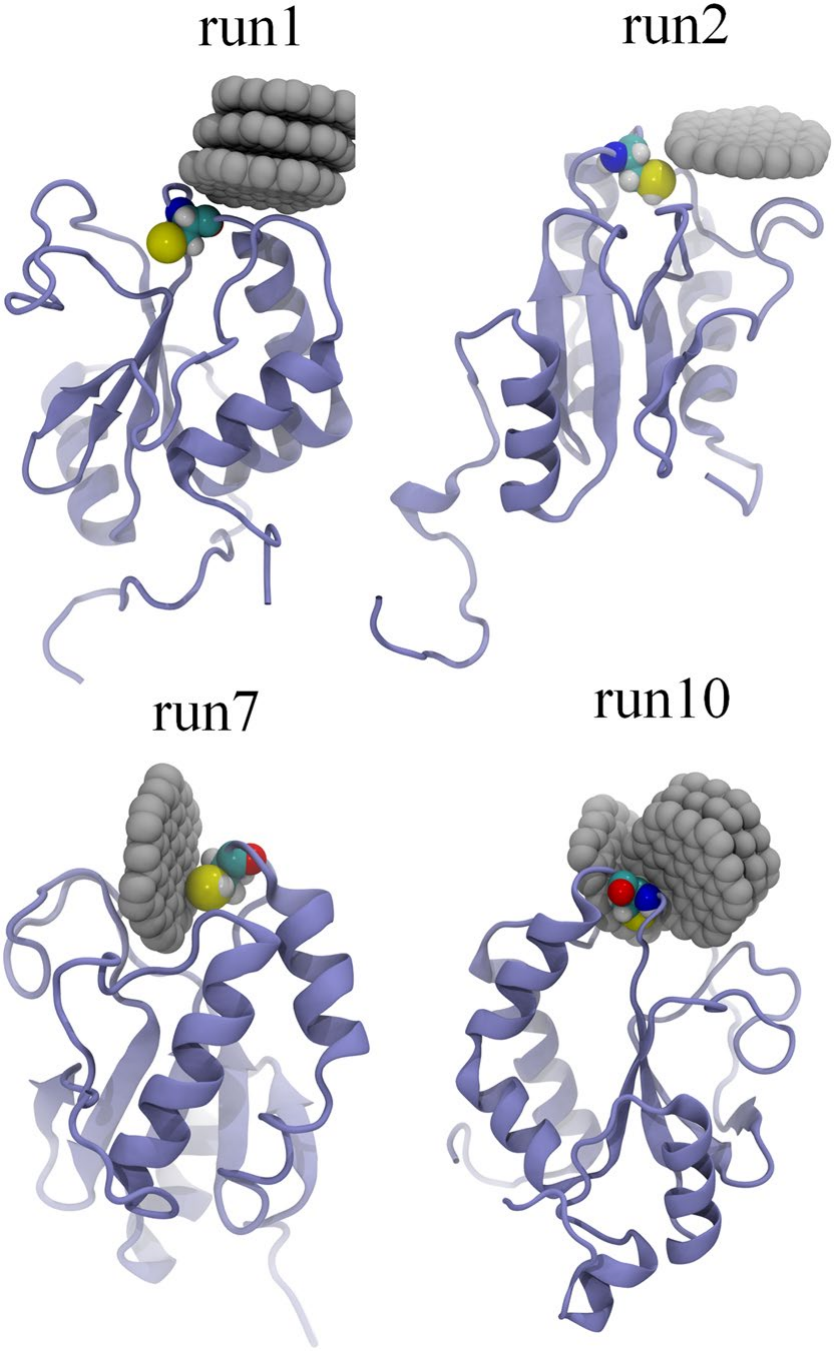
Final conformations of GQDs binding to Cys81 of AGR2 protein. Only four trajectories (i.e., run1, run2, run7 and run10) present the tight binding of GQDs to Cys81. The AGR2 is shown by iceblue ribbons, the Cys81 is displayed with cyan, red, blue and yellow spheres, and the GQDs are illustrated by gray spheres.

The final binding conformations (depicted in Fig. 2) of GQDs to the active site of AGR2 reveal two distinctive binding configurations. Herein, we just show the final conformations of the trajectories where GQDs intimately contact to Cys81 residue, because we want to observe the covering of GQDs to the active site of AGR2. In one scenario, a GQD cluster (comprising several GQD monomers, for instance, three in run1 and four in run2) adsorbs to the exposed surface of Cys81 in the solution. The alternative scenario involves an individual GQD monomer making contact with Cys81. The complete coverage of GQDs over the exposed region of Cys81 undoubtedly hampers the normal biological function of AGR2, showing its negative role in forming mixed disulfide bonds with intestinal mucin MUC2. On the other hand, the interaction of a single GQD with Cys81 may potentially interfere the normal biological interaction of AGR2 with MUC2. Interestingly, one GQD monomer usually binds to the groove comprising some loops nearby the Cys81 (e.g., run2 and run7), which is guided by the hydrophobic interaction because both GQD and this groove are hydrophobic (Fig. S2). Thus, the intimate attachment of GQDs to the active site (Cys81 amino acid) of the AGR2 protein is expected to significantly impede the normal biological function of AGR2, consequently inducing potential toxicity.

**Figure 3:**
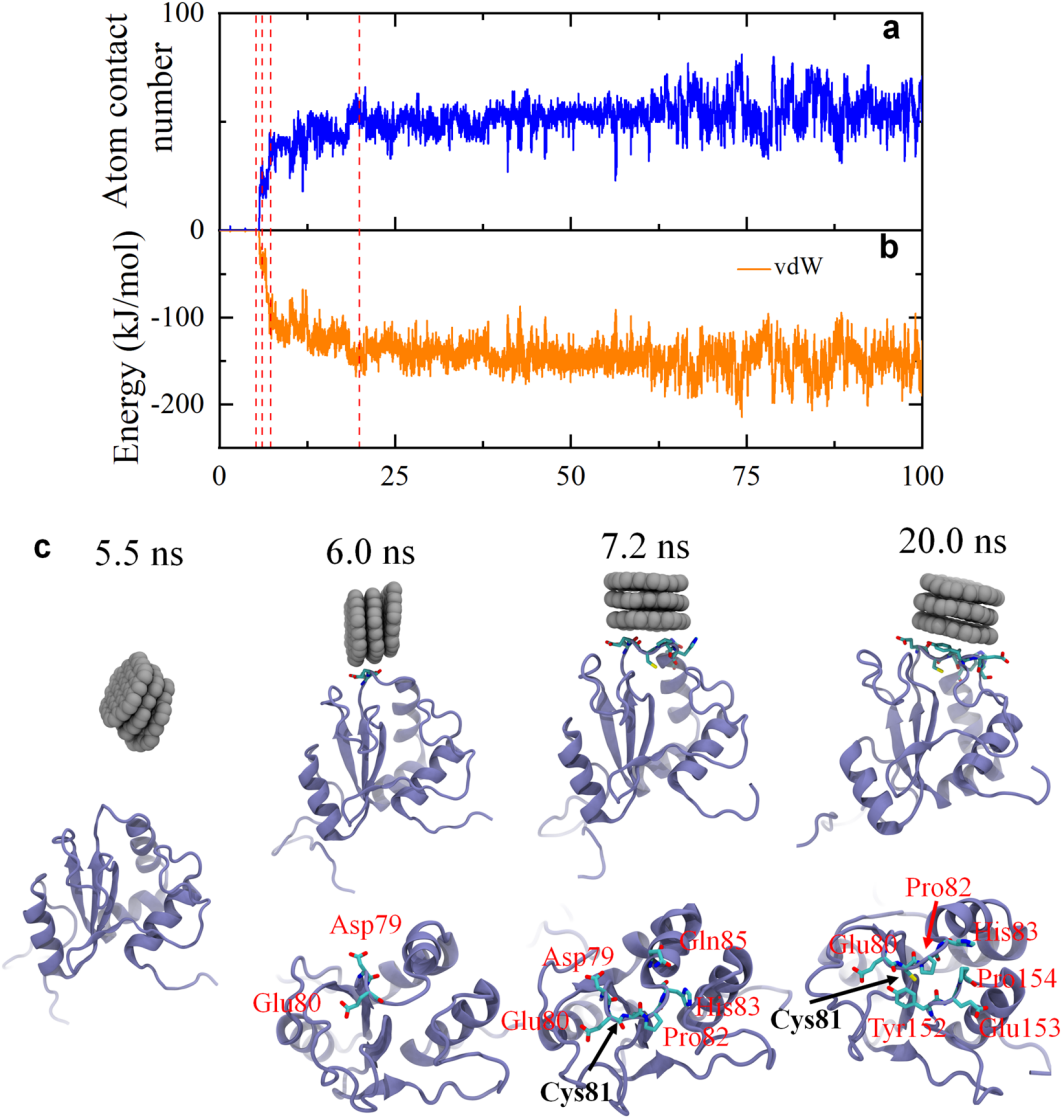
Kinetics of GQDs binding to the active site (Cys81) of AGR2 in a typical trajectory. (**a**) Time-dependent atom contact number of AGR2 to GQDs associated to the Cys81. (**b**) Time-dependent interaction energy (vdW energy) of AGR2 to GQDs associated to the Cys81. The red dashed lines denote the critical time points during the binding process. (**c**) Snapshots at critical time points. The residues related to the binding interface of GQDs and AGR2 are shown with sticks and highlighted by their residue names.

To delve deeper into the binding dynamics between GQDs and the AGR2 active site, a specific trajectory (run1) was selected to scrutinize the evolution of atom contact numbers, interaction energy, and capture several binding snapshots to vividly illustrate the binding process (Fig. 3). Initially, three GQDs aggregate to form a GQD cluster in the solution at 5.5 ns. Given the GQD's structural composition involving various benzene rings, their robust aggregation into a GQD cluster occurred in a layer-by-layer pattern, driven by the robust intermolecular forces of π-π stacking, hydrophobic, and van der Waals (vdW) interactions. At this time, the GQD cluster has yet to make contact with AGR2, resulting in a zero atom contact number and vdW energy. It was only after 0.5 ns that the GQD cluster establishes contact with the AGR2 surface, starting an intimate connection between the edges of three GQDs (the GQD cluster) and two AGR2 surface residues, Asp79 and Glu80. Simultaneously, the interfacial atom contact number and vdW interaction energy approached 20 and − 33.2 kJ/mol, respectively. Subsequently, at 7.2 ns, the GQD cluster rotates 90°, revealing a basal face of one GQD compactly aligned on the AGR2 surface. During this period, the number of contact residues increases to 6—Asp79, Glu80, Cys81, Pro82, His83, and Gln85—resulting in the atom contact number and vdW energy increasing to almost double to triple the initial values (atom contact number: 38 and vdW energy: − 107 kJ/mol). Thereafter, at 20.0 ns, the GQD cluster undergoes limited structural rearrangement, leading to the desorption of some residues (Asp79 and Glu80) and the adhesion of others (Tyr152, Glu153, and Pro154). This alteration elevates the atom contact number to 56 and the vdW energy to − 148.9 kJ/mol. Throughout the subsequent 80-ns trajectory, the interaction between the GQD cluster and AGR2 protein exhibits minor variations in the atom contact number and vdW energy. Importantly, among the contact residues, the active site, Cys81, is involved in the interface binding. Notably, the GQD cluster entirely encapsulated Cys81, rendering it fully buried in the interior, potentially causing the inactivation of the AGR2 protein function.

### GOQDs touch the active site of AGR2 protein

**Figure 4:**
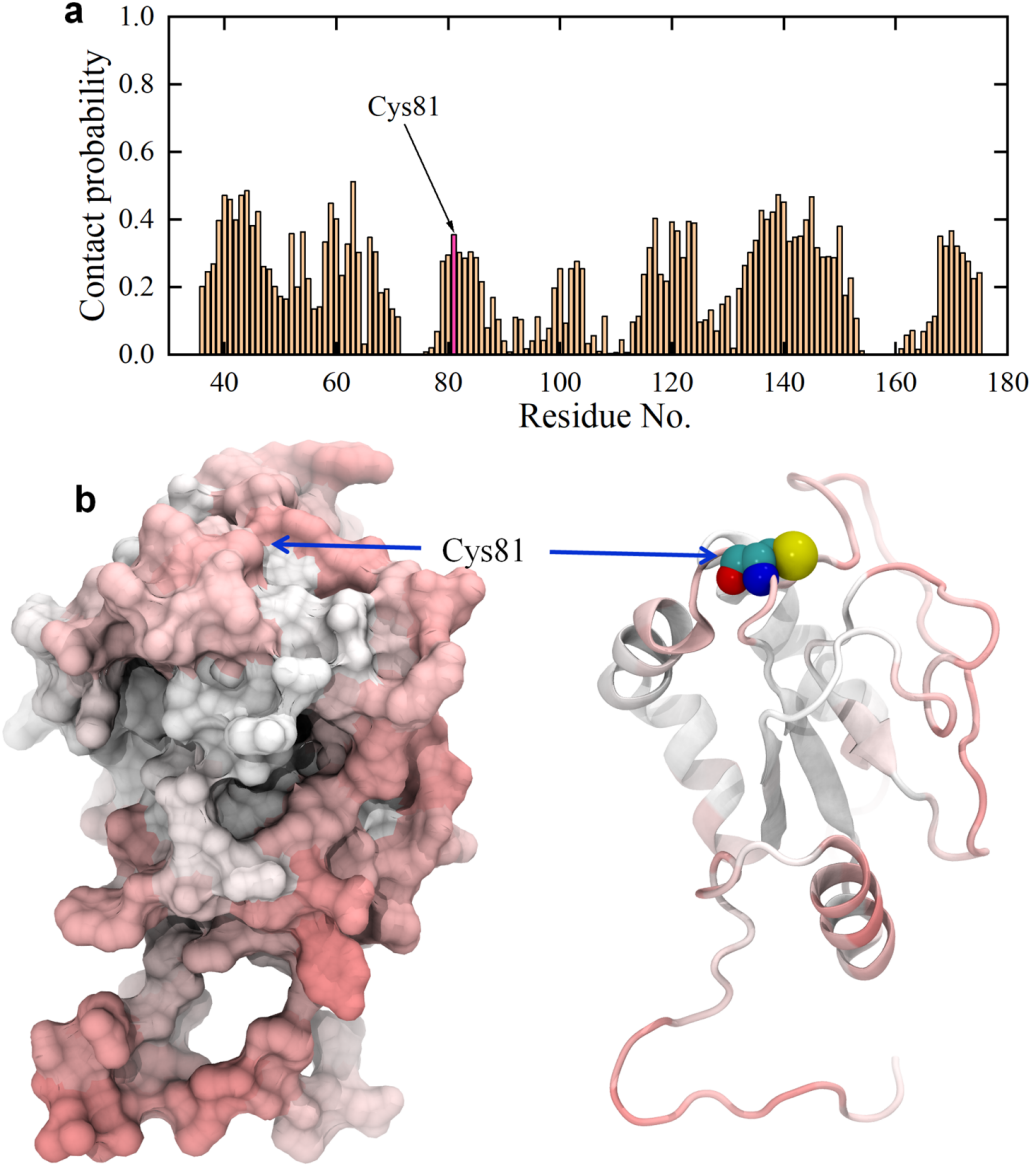
(**a**) Contact probability of GOQDs to each residue of AGR2 protein. The data are averaged from ten parallel trajectories. The contact probability of active site of AGR2, i.e., Cys81, is highlighted. (**b**) Left: Contact probability surface of AGR2; Right: Contact probability secondary structure of AGR2. The red and white colors indicate the high and low contact probabilities of GOQDs to AGR2 protein. The Cys81 is pointed by blue arrows and is shown with spheres (carbon atoms: cyan; oxygen atom: red; nitrogen atom: blue; sulfur atom: yellow) in the right picture.

**Figure 5:**
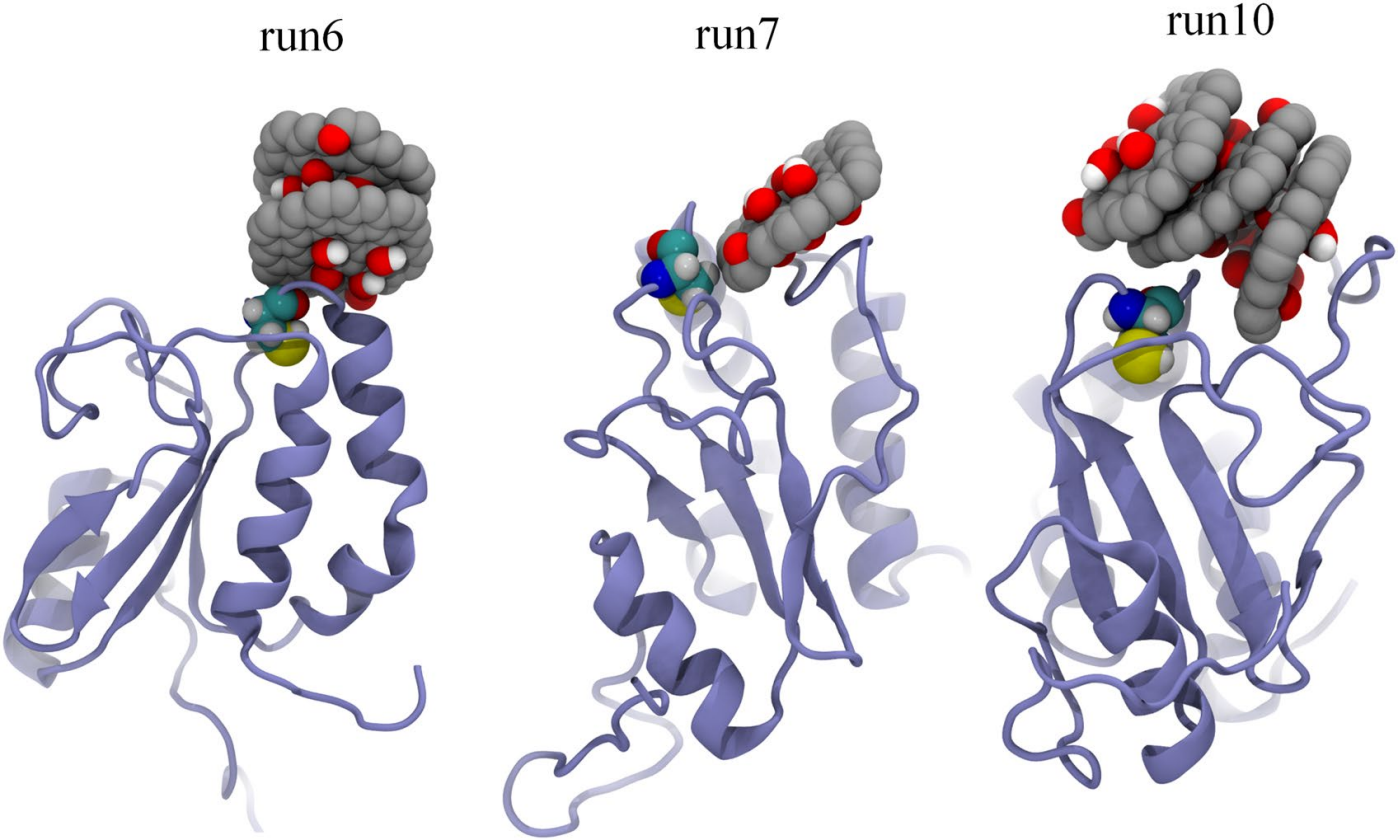
Final conformations of GOQDs binding to Cys81 of AGR2 protein. Only three trajectories (i.e., run6, run7 and run10) present the tight binding of GQDs to Cys81. The AGR2 is shown by iceblue ribbons, the Cys81 is displayed with cyan, red, blue and yellow spheres, and the GOQDs are illustrated by gray (carbon), red (oxygen) and white (hydrogen) spheres.

Next, we examined the interaction of GOQD with AGR2. Figure 4 illustrates the contact probability between each residue of the AGR2 protein and GOQDs. Numerically, Fig. 4a demonstrates that numerous AGR2 residues exhibit a substantial contact probability with GOQDs, with many exceeding 0.2. Some residues, however, present zero contact probability, suggesting a notable affinity of GOQDs for binding to the AGR2 protein surface. However, in contrast to GQDs (Fig. 1a), GOQDs exhibit a relatively lower contact probability with AGR2, indicating a diminished binding affinity. Nevertheless, the active site of AGR2 (Cys81) presents a substantial contact probability of 0.355. Therefore, GOQDs also reveal a pronounced affinity for the Cys81 site of the AGR2 protein, suggesting a potential interference with the normal biological function of AGR2 by GOQDs. Subsequently, we present the final conformations of GOQDs binding to Cys81, where three trajectories depict the robust binding of GOQDs to Cys81 (as displayed in Fig. 5). Herein, we just show the final conformations of the trajectories where GOQDs intimately contact to Cys81 residue, because we want to observe the covering of GOQDs to the active site of AGR2. Notably, these conformations involve one, two, and three GOQDs interacting with Cys81. Furthermore, in runs 6 and 10, GOQDs nearly cover the exposed region of Cys81, indicating a potential obstruction preventing Cys81 from interacting with its biological target. Conversely, in run 7, only one GOQD interacts with Cys81, potentially causing interference with the biological functionality of the active site Cys81.

**Figure 6:**
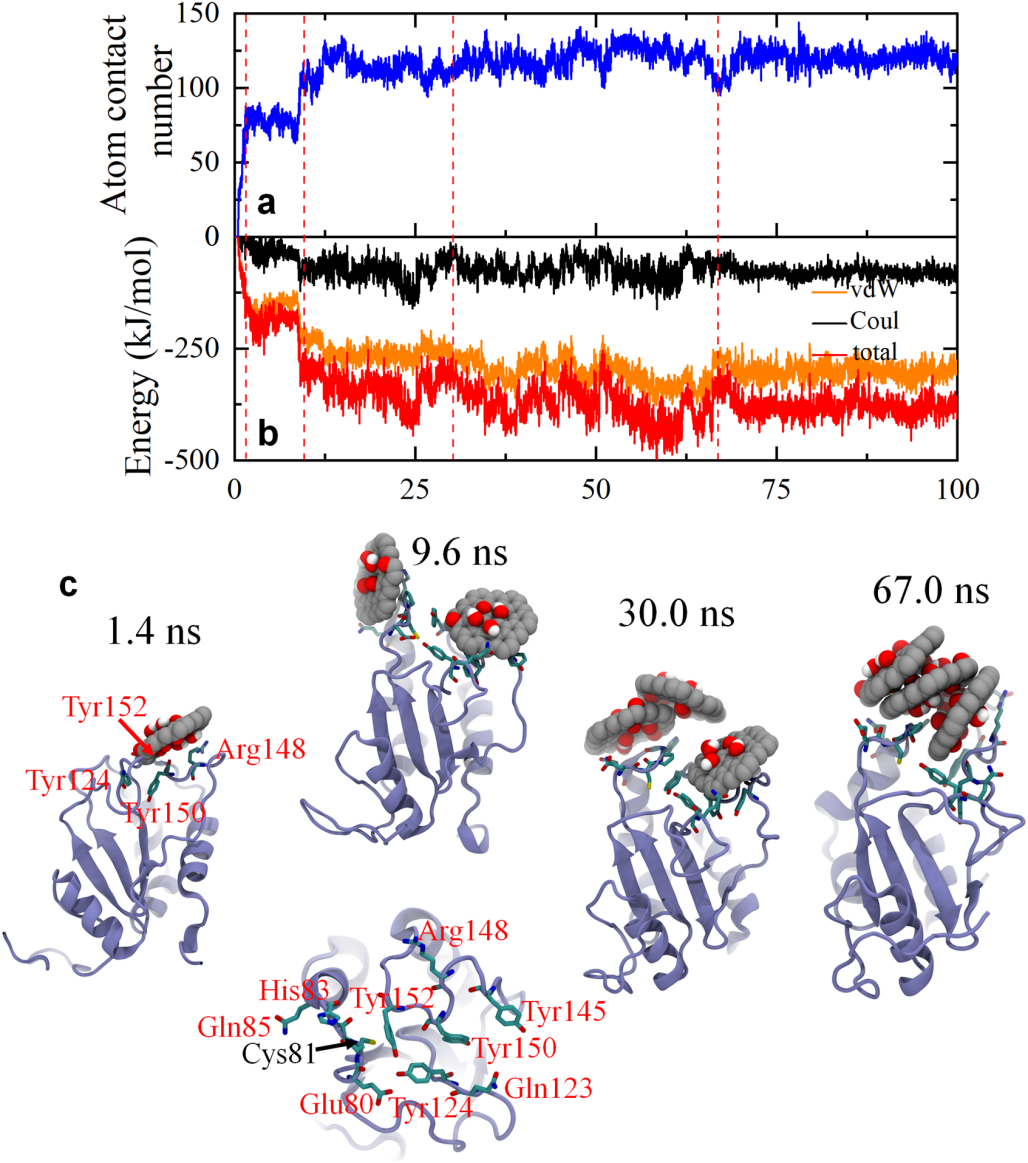
Kinetics of GOQDs binding to the active site (Cys81) of AGR2 in a typical trajectory. (**a**) Time-dependent atom contact number of AGR2 to GOQDs associated to the Cys81. (**b**) Time-dependent interaction energy (vdW energy) of AGR2 to GOQDs associated to the Cys81. The red dashed lines denote the critical time points during the binding process. (**c**) Snapshots at critical time points. The residues related to the binding interface of GOQDs and AGR2 are shown with sticks and highlighted by their residue names.

Moreover, for an in-depth exploration of the binding dynamics of GOQDs to the AGR2 active site, a representative trajectory (run10) was chosen to analyze the evolution of atom contact numbers, interaction energies, and visualize the binding process through a series of snapshots (Fig. 6). At an early time point of 1.4 ns, one GOQD initiates contact with AGR2 through four residues (Tyr124, Arg148, Tyr150, and Tyr152). This interaction causes a sharp rise in the atom contact number to 58 while decreasing the interaction energy to − 105.0 kJ/mol, with the van der Waals (vdW) and Coulomb energies separately at − 101.6 and − 3.4 kJ/mol. This highlights that the initial adsorption of a GOQD to AGR2 is primarily driven by the vdW interaction and slightly by Coulomb interaction. Subsequently, the second GOQD binds to AGR2 in proximity to the first contacted GOQD at 9.6 ns, involving additional interaction residues: Glu80, Cys81, His83, Gln85, and Gln123. This improves the atom contact number and interaction energy to 110 and − 310.7 kJ/mol, respectively, with the vdW energy significantly dominating the ratio at − 234.5 vs − 76.2 kJ/mol (vdW vs. Coulomb energy). By 30.0 ns, the third GOQD binds to a previously adsorbed GOQD but does not directly contact AGR2. Over the duration from 30.0 to 67.0 ns, the three GOQDs progressively aggregate and eventually form a layer-by-layer structure at 67.0 ns. This binding configuration remains stable on AGR2 for the remainder of the simulation time until 100 ns. Importantly, the active site Cys81 of AGR2 closely interacts with the GOQD cluster and notably, the GOQD cluster effectively encases Cys81, isolating the active site from its surrounding environment, including the targeted protein. Differing from the GQDs' binding to AGR2, the GOQDs adhere to AGR2 individually before forming the GOQD cluster. Furthermore, the obstruction of GOQDs to Cys81 is energetically facilitated by both vdW and Coulomb energies, unlike GQDs, which are primarily driven by vdW energy to bind to the active site of AGR2. Thus, both GQDs and GOQDs have the potential to directly interact with and encase the active site of AGR2 (i.e., Cys81), potentially compromising the normal biological function of this protein.

## Conclusion

In summary, we establish two typical simulation systems, including AGR2 enveloped by five GQDs or GOQDs, to investigate their potential impact. In our simulations, we chose five GQDs/GOQDs as examples, with the aim of exploring their potential influence to AGR2 protein. Through ten parallel MD simulations for each system, our results reveal that GQDs, either as individual monomers or as a clustered assembly, can adhere to the active site (Cys81) of the AGR2 protein. These adhesions lead to two significant consequences: firstly, the GQD monomer tightly interacts with Cys81, potentially disrupting the normal connection of Cys81 on AGR2 to its targeted protein; secondly, GQD clusters directly contact and envelop the Cys81 residue, causing complete burial within the interior and impeding the normal communication of AGR2 with its targeted protein. Kinetic analyses demonstrate that the adsorption of GQDs onto AGR2 is primarily facilitated by vdW interaction energy. Furthermore, GOQDs exhibit similar behavior, touching the Cys81 residue and even covering the active site, resembling the actions of GQDs. This similarity suggests that GOQDs also possess the potential to induce a toxic effect on the AGR2 protein. That is to say, GQDs and GOQDs can directly adhere on and cover the active site of AGR2 protein, which may inhibit the normally physiological binding of AGR2 active site to MUC2 (a direct involvement of AGR2 in mucin processing), finally heightening susceptibility to colitis. These findings elucidate the potential toxicity of GQDs and GOQDs towards the AGR2 protein and expound on the underlying molecular mechanisms, offering valuable insights for the safe and efficacious utilization of such nanomaterials in the biomedicine.

Although our theoretical simulations reveal that the potential blockage of GQDs/GOQDs to AGR2 protein’s active site yields the possible toxicity, the simulation setup is a very simplified model, which does not consider the complex environment in a real intestine. Therefore, the further investigation may be needed to confirm such possible biological toxicity through in vivo or in vitro experimental examinations.

## Methods

We built two typical simulation systems: one involving an AGR2 protein and five GQDs, and the other comprising an AGR2 protein with five GOQDs. Herein, we used five GQDs/GOQDs with the main aim of mimicking the effect of GQDs/GOQDs with high concentration to AGR2 protein. In addition, more GQDs/GOQDs are beneficial to observe the binding of GQDs/GOQDs to AGR2 protein. The initial distances of GQDs and GOQDs and AGR2 were set to over 1.2 nm to prevent any artificial original contact between the GQDs and AGR2. The GQDs were modeled using Lennard–Jones (LJ) particles with parameters of ε_cc_ = 0.36 kJ/mol and σ_cc_ = 0.34 nm. Force field parameters for the GOQDs were established based on previous research (details see Table S1)^41^. Both GQDs and GOQDs were established possessing the same surface dimensions. The crystal structure of AGR2 (PDB code: 2LNT)^42^ was obtained from the RCSB Protein Data Bank. Subsequently, the GQDs/AGR2 and GOQDs/AGR2 complexes were dissolved in 0.15 M NaCl solutions. It was worth noting that AGR2 protein exists in the intestine where the environment is commonly acidic milieu rather than normal salt solutions. However, we herein adopted the normal 0.15 M NaCl solution as a simplified model, because GROMACS software package cannot allow the acidic environment (i.e., requiring neutral solution).

All simulations were performed using the GROMACS (version 2018)^43^ software package. The VMD software^44^ was utilized for the analysis and visualization of the simulation results. Force fields for the protein and ions were treated with the CHARMM27 force field^45^. The TIP3P^46^ water model was employed to simulate the behavior of water molecules. The temperature was sustained at 300 K using a v-rescale thermostat^47^, and the pressure was maintained at 1 atm with a Parrinello-Rahman barostat^48^. Long-range electrostatic interactions were managed using the PME method^49^, while van der Waals (vdW) interactions were computed with a cutoff distance of 1.2 nm. All solute bonds linked to hydrogen atoms were held constant at their equilibrium values using the LINCS algorithm^50^, and the water geometry was constrained using the SETTLE algorithm^51^. During the production runs, a time step of 2.0 fs was utilized, and data were gathered every 10 ps. Each system was explored through ten independent 100 ns trajectories.

The interaction energy between GQDs/GOQDs and AGR2 protein was calculated via (1) generating the tpr file aiming at rerunning the trajectories (including groups of the GQDs contacted the active site of AGR2 and AGR2 protein), (2) rerunning the trajectories using the generated tpr file to output the ener.edr file, and (3) using the GROMACS tool, gmx energy, to generate the vdW and Coulomb energies.

### Supplementary Information

Supplementary Information.

## Data Availability

The datasets used and/or analysed during the current study available from the corresponding author on reasonable request.
