# Supplementary material for: Potential toxicity of graphene (oxide) quantum dots via directly covering the active site of anterior gradient homolog 2 protein
Source: Sci Rep. 2024 Mar 26;14:7091. doi: 10.1038/s41598-024-57677-9 (PMC10963778; doi:10.1038/s41598-024-57677-9)
Supplement: Supplementary file 1 — Supplementary Information. [file 41598_2024_57677_MOESM1_ESM.docx]

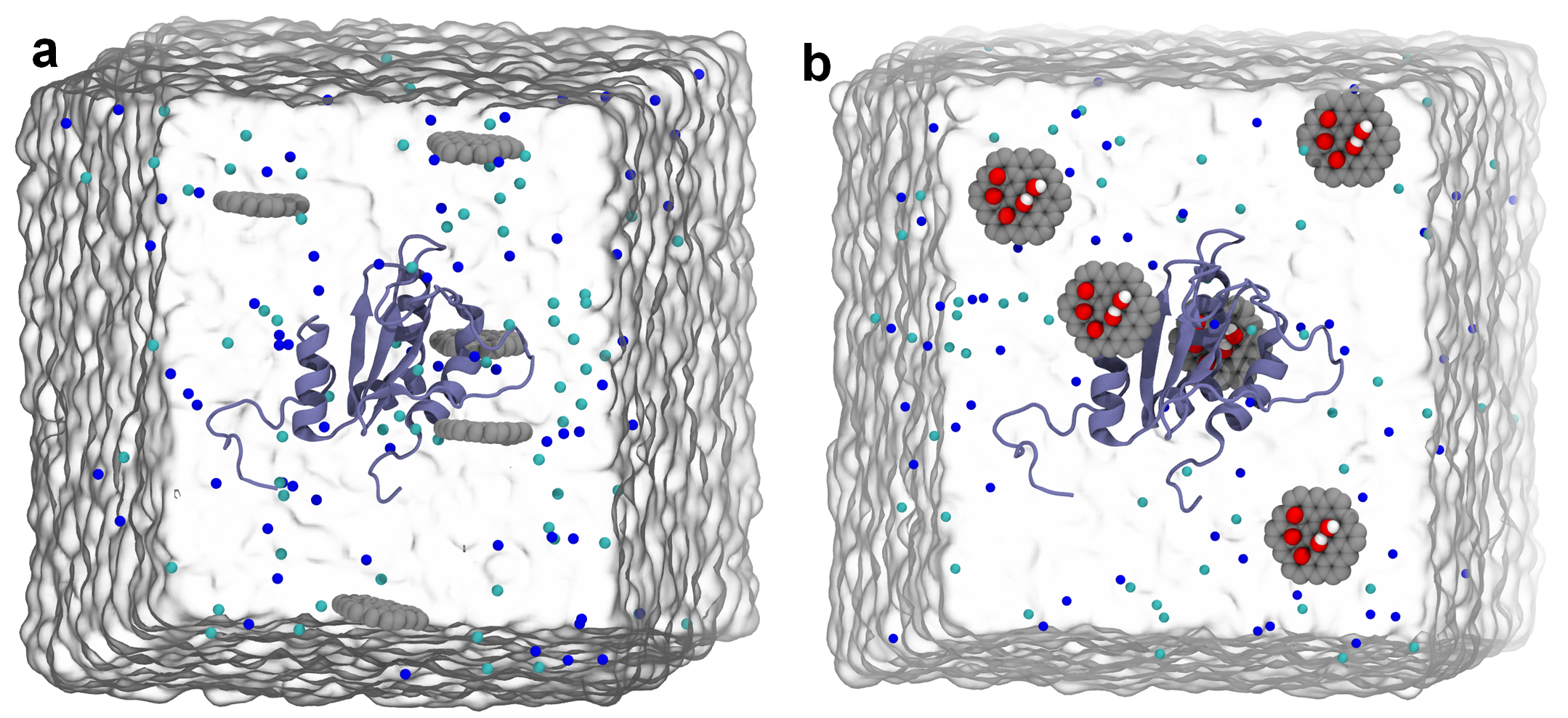


Figure S1. Initial simulation setups pf AGR2/GQDs (a) and AGR2/GOQDs.


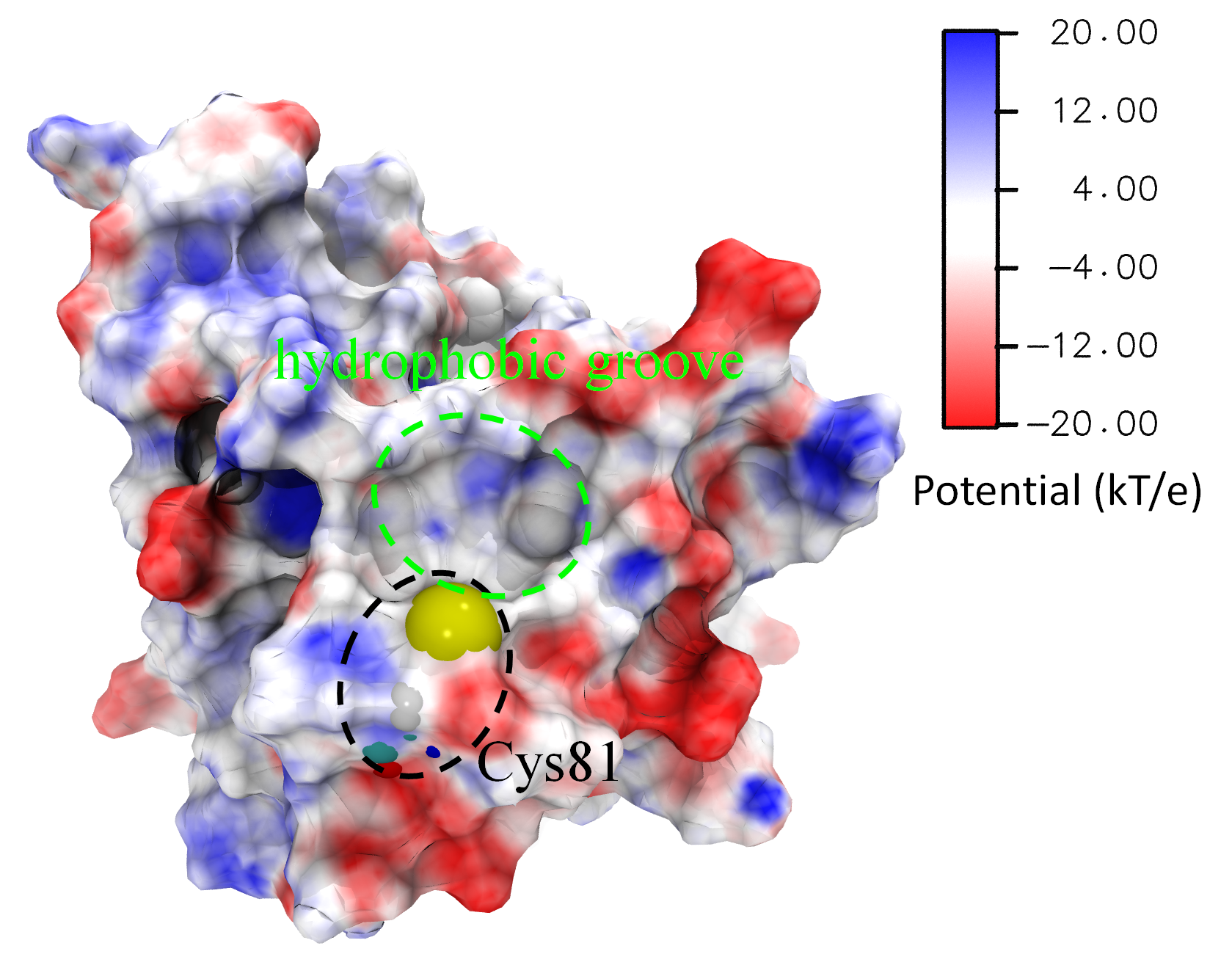


Figure S2. APBS potential surface of AGR2 protein. Cys81 is highlighted by black circle and the hydrophobic groove is denoted by green circle.

Table S1. Force field parameters of GOQD. Other carbon atoms in GOQD have the same force fields with those in GQD.

|  | σ (nm) | ε (kJ/mol) | Charge |
| --- | --- | --- | --- |
| O (hydroxyl) | 0.31 | 0.88 | -0.57 |
| H (hydroxyl) | 0 | 0 | 0.39 |
| O (epoxy) | 0.30 | 0.71 | -0.36 |
| C (connected to hydroxyl/epoxy) | 0.34 | 0.46 | 0.18 |
